# Obesity in Humans Is Characterized by Gut Inflammation as Shown by Pro-Inflammatory Intestinal Macrophage Accumulation

**DOI:** 10.3389/fimmu.2021.668654

**Published:** 2021-05-12

**Authors:** Theresa V. Rohm, Regula Fuchs, Rahel L. Müller, Lena Keller, Zora Baumann, Angela J. T. Bosch, Romano Schneider, Danny Labes, Igor Langer, Julia B. Pilz, Jan H. Niess, Tarik Delko, Petr Hruz, Claudia Cavelti-Weder

**Affiliations:** ^1^ Clinic of Endocrinology, Diabetes and Metabolism, University Hospital Basel, Basel, Switzerland; ^2^ Department of Biomedicine (DBM), University of Basel, University Hospital Basel, Basel, Switzerland; ^3^ Department of Surgery, University Hospital Basel, Basel, Switzerland; ^4^ Clarunis, Department of Visceral Surgery, University Center for Gastrointestinal and Liver Diseases Basel, St. Clara Hospital and University Hospital Basel, Basel, Switzerland; ^5^ Department of Visceral Surgery, Lindenhof Hospital, Bern, Switzerland; ^6^ AMB-Arztpraxis MagenDarm Basel, Basel and MagenDarm Aarau, Aarau, Switzerland; ^7^ Department of Endocrinology, Diabetology and Clinical Nutrition, University Hospital Zurich (USZ) and University of Zurich (UZH), Zurich, Switzerland

**Keywords:** Obesity, diabetes, metabolic disease, chronic inflammation, intestinal inflammation, mucosal immunity, macrophages, monocytes

## Abstract

Chronic low-grade inflammation is a hallmark of obesity and associated with cardiovascular complications. However, it remains unclear where this inflammation starts. As the gut is constantly exposed to food, gut microbiota, and metabolites, we hypothesized that mucosal immunity triggers an innate inflammatory response in obesity. We characterized five distinct macrophage subpopulations (P1-P5) along the gastrointestinal tract and blood monocyte subpopulations (classical, non-classical, intermediate), which replenish intestinal macrophages, in non-obese (BMI<27kg/m^2^) and obese individuals (BMI>32kg/m^2^). To elucidate factors that potentially trigger gut inflammation, we correlated these subpopulations with cardiovascular risk factors and lifestyle behaviors. In obese individuals, we found higher pro-inflammatory macrophages in the stomach, duodenum, and colon. Intermediate blood monocytes were also increased in obesity, suggesting enhanced recruitment to the gut. We identified unhealthy lifestyle habits as potential triggers of gut and systemic inflammation (i.e., low vegetable intake, high processed meat consumption, sedentary lifestyle). Cardiovascular risk factors other than body weight did not affect the innate immune response. Thus, obesity in humans is characterized by gut inflammation as shown by accumulation of pro-inflammatory intestinal macrophages, potentially *via* recruited blood monocytes. Understanding gut innate immunity in human obesity might open up new targets for immune-modulatory treatments in metabolic disease.

## Introduction

As a major interface to the external environment, the gut is continuously exposed to antigens derived from food and gut microbiota (1). This exposure is countered by immune cells accumulating in the intestinal mucosa as part of the immune barrier. Macrophages are the most abundant leukocytes in the healthy gut (2). They originate from blood monocytes that continuously enter the mucosa in a CCR2-dependent manner (3). Monocytes in the blood can be divided in three major populations with distinct functions: non-classical monocytes patrol the vasculature, classical monocytes have an immediate response in inflammation, while intermediate monocytes have a delayed response (4). Classical monocytes have been shown to give rise to intestinal macrophages, which differentiate *via* several intermediary subpopulations to mature intestinal macrophages, a process that has become known as “waterfall” (5, 6).

Five intestinal macrophage subpopulations have been described along this differentiation trajectory in both mice and humans (5, 6). First, extravasated monocytes differentiate into pro-inflammatory intestinal macrophage subpopulations P1 and P2, and then into an intermediate subpopulation P3. These intermediaries then give rise to anti-inflammatory/resident macrophages, which include subpopulations P4 and P5 (5–7). The differentiation is estimated to take approximately 5–6 days, with mature macrophages having a half-life of 6–8 weeks (8). Mature intestinal macrophages have many functions: they have been described as maintaining survival and expansion of FoxP3+ regulatory T cells (9, 10), which are crucial for tolerance of orally ingested antigens. Moreover, their avid phagocytic activity and distinct anergy towards inflammatory stimuli enables them to act as efficient scavengers without inducing inflammation (5, 11, 12).

In the healthy gut, most intestinal macrophages consist of the anti-inflammatory/resident subpopulations (5). This steady state is perturbed in inflammatory bowel disease (IBD), such as Crohn’s disease and ulcerative colitis (1, 5), where macrophages retain their immature pro-inflammatory phenotype, rather than further differentiating into anti-inflammatory/resident intestinal macrophages (5, 6, 13). This observation indicates that in sterile inflammation, pro-inflammatory intestinal macrophage subpopulations are pathogenic and drive inflammation and tissue damage in IBD patients (1). Also, in infectious disease mouse models, pro-inflammatory intestinal macrophage subpopulations have been shown to increase, although they seem to play a beneficial role there in host defense (14, 15).

While human intestinal macrophages have been studied in the context of infectious or inflammatory bowel disease, their role in metabolic disease is largely unexplored. So far, macrophages have been established in adipose tissue as major players in the inflammatory process associated with metabolic disease (16). However, an inflammatory response in the gut seems to precede adipose tissue inflammation in diet-induced obesity (17). Previous studies indicate that innate gut immunity is involved in metabolic disease as mice fed a high-fat diet (HFD) have elevated levels of TLR4, TNF-alpha, and NF-kappaB in the gut wall (18, 19), and an increased inflammatory tone of intestinal macrophages (17). Moreover, when assessing intestinal macrophage subpopulations in mice fed a HFD, we found increased pro-inflammatory macrophages in the gut (20). This is similar to what has been described in IBD and suggests disrupted differentiation of intestinal macrophages towards an anti-inflammatory/resident state. As colon-specific macrophage depletion in mice fed a HFD improved glucose tolerance and insulin sensitivity in mice, colonic macrophages seem to be causally linked to glycemic control (20). However, a comprehensive analysis of intestinal macrophage subpopulations in human obesity has been missing so far.

The aim of our study was to characterize human intestinal macrophages in non-obese and obese subjects at different anatomical locations along the gastrointestinal tract. Additionally, we sought to determine whether and how body composition, different lifestyle behaviors (nutrition and physical activity), and disease variables impact gut innate immunity. Understanding the role that mucosal innate immunity plays in human obesity might open up new possibilities for immune-modulatory treatments in metabolic disease.

## Methods

### Human Subjects

Subjects were recruited at Clarunis, University Center for Gastrointestinal and Liver Diseases Basel, the doctor’s office MagenDarm Basel, or the Lindenhof Hospital Bern. They were planned for bariatric surgery, colorectal cancer screening (recommended in Switzerland after the age of 50 years for individuals without family exposure), or a diagnostic gastroscopy or colonoscopy on the basis of symptoms suggestive for gastroesophageal reflux disease, gastritis, or irritable bowel syndrome.

Colon biopsies were collected from obese (BMI >32 kg/m^2^, n=10) and non-obese (BMI <27 kg/m^2^, n=13) individuals of both genders, and gastric biopsies were collected from obese (BMI >32 kg/m^2^ or BMI >35 kg/m^2^ for subjects undergoing bariatric surgery, n=12) and non-obese (BMI <27 kg/m^2^, n=12) individuals of both genders. Jejunum samples were obtained from obese patients undergoing bariatric surgery (BMI >35 kg/m^2^, n=6). Three subjects included in the non-obese group had slightly higher BMI levels than 27 kg/m^2^ (27.8 kg/m^2^, 28.0 kg/m^2^ and 28.5 kg/m^2^). Blood serum and EDTA blood was obtained from obese (n=25) and non-obese patients (n=21).

Exclusion criteria were inability to provide informed consent, active smoking, intake of corticosteroids, anti-inflammatory/immunosuppressive drugs potentially altering immune cells, clinical signs of current infection, known anemia (e.g. hemoglobin < 110 g/L for males, < 100 g/L for females) or neutropenia (e.g. leukocyte count < 1.5 × 10^9/L or ANC < 0.5 × 10^9/L), known immunodeficiency (e.g. HIV), vasculitis or collagenosis, inflammatory bowel disease, adrenal insufficiency and/or substitution with glucocorticoids, known clinically significant kidney or liver disease (e.g. creatinine > 1.5 mg/dL, ASAT/ALAT > 2 × ULN, alkaline phosphatase > 2 × ULN, or total bilirubin [tBili] > 1.5 × ULN, liver cirrhosis Child B or C), risky daily alcohol consumption (> 24g/d for males, > 12g for females), known uncontrolled congestive heart failure, known uncontrolled malignant disease or currently pregnant or breastfeeding. Researchers were blinded during sample-processing and data acquisition. For the statistical analysis between obese and non-obese patients, researchers were unblinded.

### Study Approval

Patients provided written informed consent. The study was performed according to the case report form, and human material was collected and used with informed consent. Permission for the human study (ID: 2018-00712; full title “Characterization of human intestinal macrophages in metabolic disease- iMAC study” https://clinicaltrials.gov/) was obtained by the Ethical Commission in Basel (Ethikkommission Nordwest- und Zentralschweiz).

### Isolation of Intestinal Macrophages

Gastrointestinal tissue was collected from non-obese (BMI <27 kg/m^2^) and obese (BMI >32 kg/m^2^ or BMI >35 kg/m^2^ for subjects undergoing bariatric surgery) subjects undergoing screening colonoscopies (colon transversum biopsy), gastroscopy (stomach and duodenum biopsy), or bariatric surgery (jejunum piece). To isolate lamina propria cells, the fat was removed from tissue specimen and in case of tissue from bariatric surgeries cut into smaller pieces. Thereafter, the epithelial layer was shaken off by washing tissue pieces twice in HBSS/2 mM EDTA 20 min at 37°C. After washing twice in HBSS, gut tissue was transferred into a gentle MACS C-tube (#130-096-334, Miltenyi Biotec) containing Complete IMDM Medium (1x IMDM, 10 % FBS, P/S, Glutamax) (3mL for biopsy samples, appropriate higher volume for bigger jejunum samples). Next, same volume of 2x Collagenase VIII (#C2139, Sigma-Aldrich) digestion solution (Complete IMDM, 2 mg/mL Collagenase VIII, 25 µg/mL DNase I) was added to start enzymatic digestion by shaking at 37°C (human biopsies: 35-40 min; jejunum 30-50 min). Later, digested tissue was homogenized by using the gentleMACS Octo Dissociator (Militenyi Biotec; program: ms_intestine-01). Digestion was stopped by adding 1% EDTA. For bariatric samples (jejunum), leukocytes were enriched by percoll gradient (40%/70%, #GE17-0891-01, GE Healthcare) and centrifugated (600 g, 20 min, 22°C, brake and acceleration 0 or 1). The lymphocyte ring was collected in FACS Buffer (1xDPBS, 0.5 % BSA, 5 mM EDTA) and washed (550 g, 5 min, 22°C). Finally, the cells were resuspended in 200 µL FACS Buffer (DPBS/0.5 % BSA/5 mM EDTA) containing Fc Blocking, and then filtered through a 35 µM strainer FACS tube (#352235, Corning).

### Isolation of Peripheral Blood Mononuclear Cells (PBMCs)

To isolate peripheral blood mononuclear cells, blood was collected in EDTA containing tubes and 1 serum tube. After dilution with DPBS (1 part blood; 3 parts DPBS), a ficoll density gradient was performed by using Lymphoprep density gradient medium (#07851, Stemcell technologies) and Leucosep tubes (#871346, OpoPharma) for 25 min (453 g, 22°C, brake: 1, acceleration: 4). The Lymphoprep layer was washed with FACS Buffer, and Red Cell Lysis Buffer (154mM NH_4_Cl, 10mM KHCO_3_, 0.1 mM EDTA) was used to remove residual erythrocytes. The remaining cells were used for further flow cytometry staining.

### Antibodies and Flow Cytometry

To reduce unspecific binding, the Fc receptor was blocked with CD16/CD32 prior to incubation with monoclonal antibodies (mAbs) for 30 min-1 h on ice. All mAbs used for flow cytometry were listed in the Key Resources Table (**Supplementary Table 1**). Samples were acquired with a BD LSRII Fortessa (BD) and analyzed with FlowJo software 10.6.1 (BD).

Human intestinal macrophage subpopulations were identified as positive for CD45 expression and negative for various lineage markers (including CD19, CD3, CD56, CD20, TCR). DCs were excluded by the absent expression of CD33 and CD64. Macrophages subsets were identified according their expression of CD14 and HLA-DR expression. Monocyte-derived macrophages were characterized as CD14^high^ (P1, P2, P3) and resident macrophages as CD14^low^ (P4, P5). Further distinction of subpopulations was done by different expression of HLA-DR, CD163 and CD209 into pro-inflammatory P1 (CD163^low^HLA-DR^low^), P2 (CD163^low^HLA-DR^inter^), intermediate P3 (CD163^high^HLA-DR^high^) and resident, anti-inflammatory subpopulations P4 (CD209^low^HLA-DR^inter^), P5 (CD209^high^HLA-DR^high^) (adapted from gating strategy (5), see **Figure 2A** and **Supplementary Figure 1A**). For further details of marker expression of P1-P5 see **Supplementary Figure 2**. Blood monocyte subpopulations were distinguished based on the differential expression of CD14 and CD16 cell surface markers (CD14^high^CD16^-^ classical, CD14^high^CD16^inter^ intermediate, and CD14^low^CD16^high^ non-classical monocytes (21); see gating strategy in **Figure 3C** and **Supplementary Figure 1B**).

### Laboratory Blood Values

Liver enzymes, such as ASAT and ALAT, blood lipids, such as cholesterol, HDL and triglycerides, and CRP were measured in plasma on the c502/c702 modules of the Cobas 8000 series from Roche Diagnostics (Roche Diagnostics, Basel, Switzerland) according to the manufacturer’s instructions.

### Statistical Analysis

The data are presented as mean ± standard error of the mean (SEM), with the numbers (n) of subjects indicated in the figure legends. To test statistical differences between two groups, an unpaired Mann-Whitney U test with two tailed distribution was run with Prism8 software (GraphPad Software, San Diego, CA). For correlation analysis, Spearman correlation was performed. The correlation coefficients (*r*) and *p* values were calculated and are included in the figures. For correlation analysis of continuous data, a linear regression line was added for a better visualization and values varying more than Mean+2SD were excluded. To adjust for BMI, we performed stratification by non-obese or obese status and multiple linear regression analysis adjusted for BMI. *P* values of 0.05 or less were considered to be statistically significant.

## Results

### Baseline Characteristics

**Figure 1:**
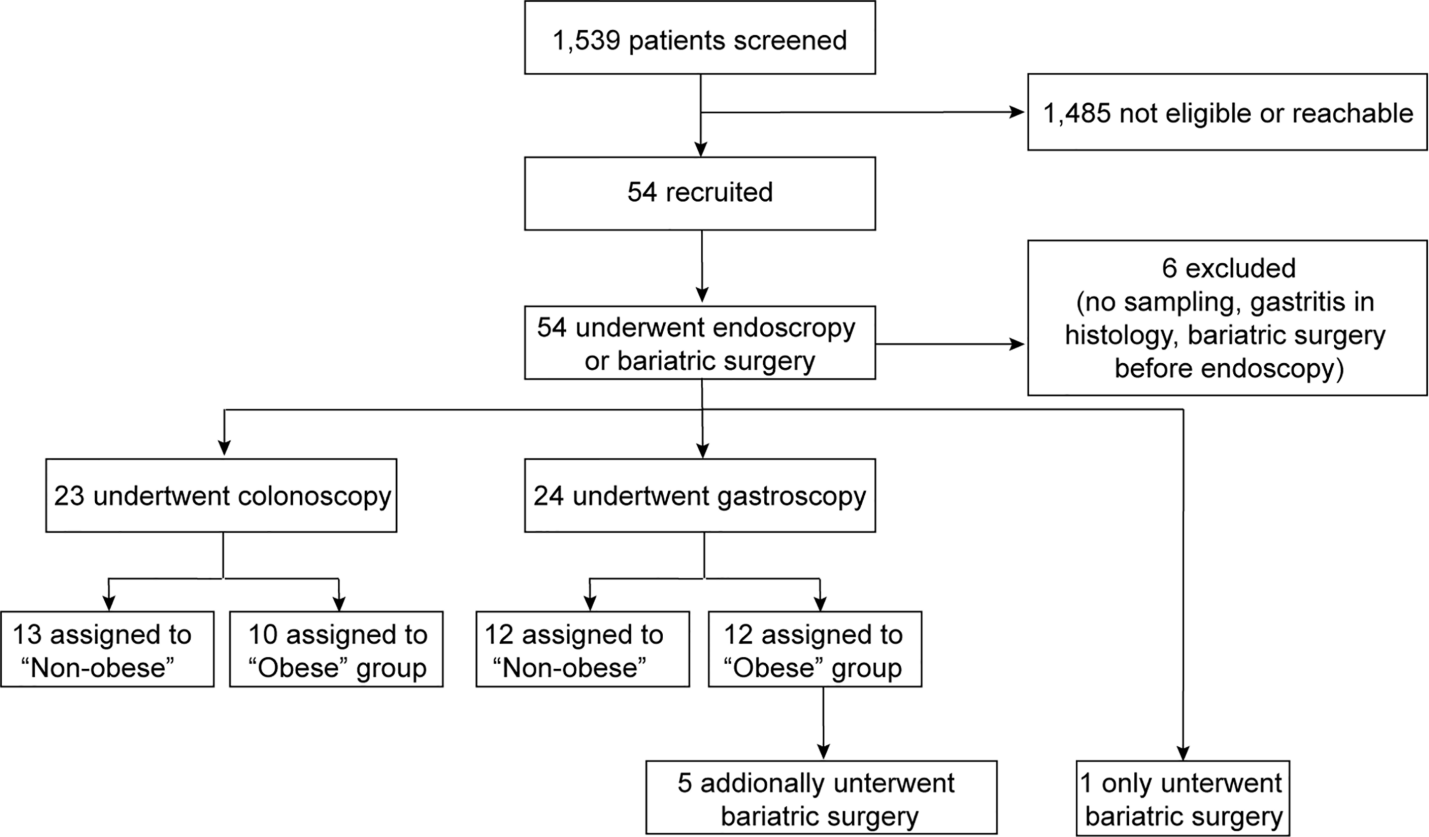
Enrollment flow by intervention group. A total of 54 study participants were recruited and the samples of 48 subjects were analyzed. Colon biopsy specimens were obtained from 13 non-obese and 10 obese individuals. Stomach and duodenum biopsies were derived from 12 non-obese and obese subjects, each. Jejunum samples were obtained from a subgroup of 6 obese individuals with a BMI >35 kg/m^2^ at the time of bariatric bypass surgery.

Fifty-four of 1,539 subjects who underwent initial screening were included in this prospective, single-center, observational study (**Figure 1**). Study participants were classified according to their body mass index (BMI) as either non-obese (BMI <27 kg/m^2^) or obese (BMI >32 kg/m^2^ for subjects undergoing colonoscopy and >35 kg/m2 for patients undergoing gastroscopy before bariatric surgery, respectively). Biopsy specimens were obtained from 13 non-obese and 10 obese individuals undergoing colonoscopy (colon biopsies), and from 12 non-obese and 12 obese subjects undergoing gastroscopy (stomach and duodenum biopsies; **Figure 1**). Jejunum samples were obtained from a subgroup of 6 obese individuals with a BMI >35 kg/m^2^ at the time of bariatric bypass surgery. Biopsy samples that were identified with active gastric inflammation by histology or were tested positive for *Helicobacter pylori* were excluded from the analysis. **Table 1** shows the baseline characteristics for the study participants stratified by non-obese or obese status. **Supplementary Table 2** further groups patients according to their intervention groups. As expected, the non-obese and the obese group differed significantly in terms of BMI, weight, waist circumference, waist-to-hip ratio (WHR), waist-to-height ratio (WHtR), C-reactive protein (CRP), alanine aminotransferase (ALT), triglycerides, and high-density lipoprotein (HDL) (**Table 1**).

**Table 1:** Baseline characteristics of study participants.

Characteristic		Non-obese (n=26)	Obese (n=25)	p-value
**Samples**	Gastroscopy (%)	12 (46)	12 (48)	
	Colonoscopy (%)	13 (50)	10 (40)	
	Blood (%)	21 (81)	24 (96)	
	Bariatric surgery (%)	0 (0)	6 (24)	
**Age (years)**		54.6 ± 3.2	50.8 ± 3.4	0.42
**Sex**				
**Male (%)**	Male (%)	13 (50)	13 (52)	1.00
	Female (%)	13 (50)	12 (48)	
**Ethnicity**	Caucasian (%)	23 (88)	23 (92)	1.00
	Other (black, asian, hispanic) (%)	3 (12)	2 (8)	
**Body weight parameters**	BMI (kg/m^2^)	23.4 ± 0.7	38.8 ± 1.2	<0.001
	Weight (kg)	69.2 ± 2.3	119.3 ± 6.1	<0.001
	Waist circumference (cm)	90.3 ± 2.4	126.6 ± 3.4	<0.001
	WHR	0.96 ± 0.01	1.01 ± 0.02	0.01
	WHtR	0.54 ± 0.01	0.72 ± 0.02	<0.001
**cvRF**	Diabetes mellitus (%)	0 (0)	5 (20)	0.02
	Hypertension (%)	8 (31)	14 (56)	0.09
	Dyslipidemia (%)	9 (35)	8 (32)	1.00
	Positive family history (%)	3 (12)	2 (8)	1.00
**Laboratory parameters**	CRP (mg/l)	1.6 ± 0.3	6.3 ± 1.2	<0.001
	ASAT (U/l)	23.6 ± 2.2	24.5 ± 1.3	0.43
	ALAT (U/l)	26.2 ± 2.8	37.3 ± 3.5	0.02
	Cholesterol (mmol/l)	4.7 ± 0.2	4.8 ± 0.2	0.80
	Triglycerides (mmol/l)	1.2 ± 0.2	1.6 ± 0.2	0.02
	LDL (mmol/l)	2.6 ± 0.2	2.8 ± 0.2	0.43
	HDL (mmol/l)	1.6 ± 0.1	1.3 ± 0.1	0.01

Data were expressed as mean ± standard error of the mean (SEM) and number (%). WHR was calculated waist circumference divided by hip circumference, and WHtR was calculated waist circumference divided by height. BMI, Body mass index; WHR, Waist-to-Hip Ratio; WHtR, Waist-to-Height Ratio; cvRF, cardiovascular risk factors; CRP, C-reactive protein; ASAT, Aspartate Aminotransferase; ALAT, Alanine Aminotransferase; LDL, Low-density lipoprotein; HDL, High-density lipoprotein.

### Intestinal Macrophages Increase Along the Gastrointestinal Tract, Particularly Anti-Inflammatory/Resident Subpopulation P5

**Figure 2:**
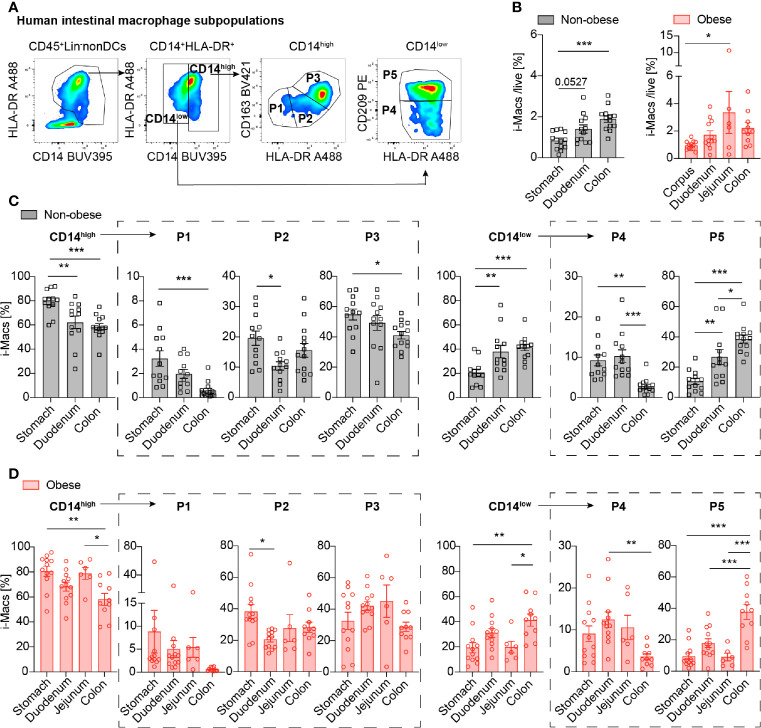
Intestinal macrophages increase along the gastrointestinal tract, particularly the anti-inflammatory/resident subpopulation P5. **(A)** Flow cytometry gating strategy of human intestinal macrophages (CD14^high^ (pro-inflammatory P1, P2 and intermediate P3) and CD14^low^ (anti-inflammatory/resident P4, P5 subpopulations). **(B)** Frequency of total intestinal macrophages in non-obese (BMI <27 kg/m^2^) and obese subjects (BMI >32 kg/m^2^) in the stomach, duodenum, and colon (n=12-13). **(C, D)** Distribution of intestinal macrophage subpopulations in non-obese **(C)** or obese subjects **(D)** in the stomach, duodenum, jejunum, and colon (n = 10-12). One data point represents one subject. Statistical data are expressed as mean ± SEM. * *p* <0.05. ** *p* <0.01, *** *p* <0.001, tested by ordinary one-way ANOVA.

We first addressed the question whether intestinal macrophages and their subpopulations P1 to P5 vary in their frequency depending on the anatomical location as antigenic exposure to foods and commensal microbiota change along the gastrointestinal tract (22). Therefore, intestinal macrophages and their subpopulations P1-P5 were analyzed by flow cytometry (**Figure 2A**, **Supplementary Figures 1, 2**) (5). The frequency of total intestinal macrophages increased along the gastrointestinal tract in both non-obese and obese individuals (**Figure 2B**). CD14^high^ macrophages including subpopulations P1/P2 (pro-inflammatory) and P3 (intermediate stage) decreased along the gastrointestinal tract (**Figures 2C, D**, left panel), whereas anti-inflammatory/resident CD14^low^ macrophages (particularly, its main subpopulation P5) increased from the stomach to the duodenum and colon (**Figures 2C, D**, right panel). In sum, intestinal macrophages and their subpopulations were found to be increasing in frequency towards the distal part of the gastrointestinal tract. This was especially true for the anti-inflammatory/resident intestinal macrophage subpopulation.

### Pro-Inflammatory Macrophages Increase Throughout the Gastrointestinal Tract in Obese Individuals and Are Accompanied by Higher Intermediate Blood Monocytes

**Figure 3:**
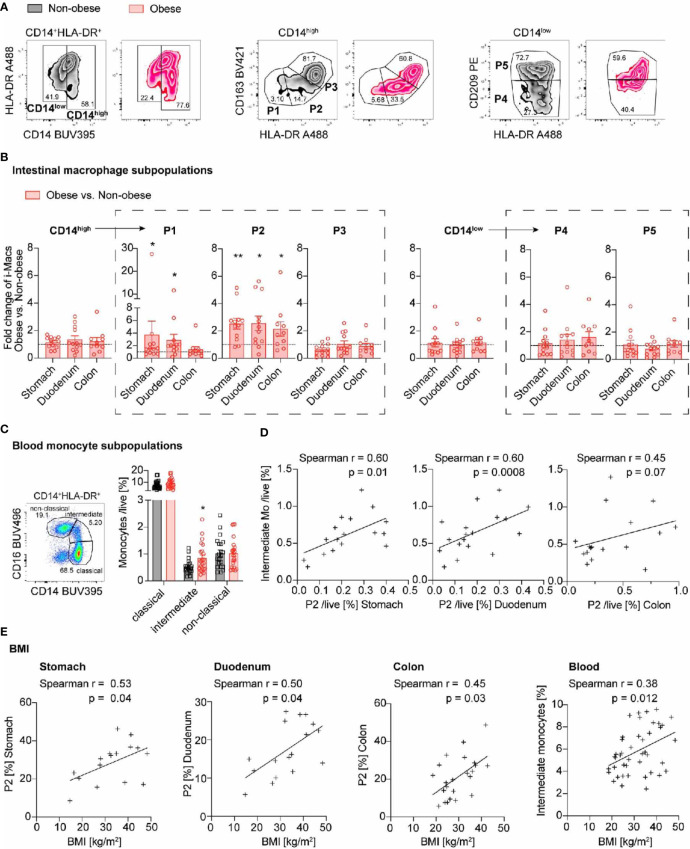
Pro-inflammatory macrophages increase throughout the gastrointestinal tract in obese individuals and are accompanied by higher intermediate blood monocytes. **(A)** Representative flow cytometry plots identifying intestinal macrophages subpopulations in non-obese (black) and obese (red) individuals. CD14^high^ represent pro-inflammatory P1, P2 and intermediate P3 subpopulations and CD14^low^ anti-inflammatory/resident P4, P5 subpopulations. **(B)** Fold change of intestinal macrophage subpopulations comparing obese (n = 10-12) to non-obese (n = 12-13) subjects along the gastrointestinal tract. **(C)** Frequency of classical, intermediate, and non-classical blood monocytes in non-obese (black; n = 22) and obese (red; n = 25) subjects. **(D)** Spearman correlation between intestinal macrophage subpopulation P2 in stomach, duodenum or colon and intermediate blood monocytes. **(E)** Spearman correlation of intestinal macrophage subpopulation P2 in stomach, duodenum, and colon transversum or intermediate blood monocytes with BMI. One data point represents one subject. Statistical data are expressed as mean ± SEM. * *p* <0.05. ** *p* <0.01, unpaired Mann-Whitney U test with two tailed distribution **(B, C)**. For Spearman correlation analysis, values above Mean + 2SD were excluded **(D, E)**.

Previously, we found that pro-inflammatory intestinal macrophage subpopulations P1 and P2 are increased in the colon of mice fed a HFD (20). It is currently unknown whether this innate immune response only occurs in the colon, or also in other parts of the gastrointestinal tract. Therefore, we compared the frequency of intestinal macrophages and their subpopulations in obese and non-obese individuals in different parts of the gastrointestinal tract (stomach, duodenum, jejunum and colon transversum). We found that obese individuals had increased proportions of the CD14^high^ pro-inflammatory macrophage subpopulation P2 in all examined parts of the gastrointestinal tract, compared to non-obese individuals (stomach 2.53 ± 1.34-fold, duodenum 2.56 ± 1.83-fold, colon 2.14 ± 1.67-fold, **Figures 3A, B**; frequencies are presented in **Supplementary Figure 3A**). In addition, the pro-inflammatory subpopulation P1 was increased in the stomach and duodenum of obese subjects (stomach 3.74 ± 7.54-fold, duodenum 2.88 ± 3.15-fold, not significant in the colon with a 1.39 ± 1.39-fold increase, **Figures 3A, B**). All other subpopulations in non-obese and obese individuals were comparable in frequency.

Leukocytes and CD103+ dendritic cells (DCs) were increased in the duodenum of obese subjects, but not in the stomach or colon, when compared to non-obese controls (**Supplementary Figures 3B, C**). Additionally, *Tnfa*, *Ccl18* and *Ccl2* showed a trend towards increased expression in duodenum of obese subjects when compared to non-obese (**Supplementary Figure 3D**).

As pro-inflammatory intestinal macrophages are constantly replenished by circulating blood monocytes (12), we asked whether this increase in pro-inflammatory intestinal macrophage subpopulations in obese subjects could be due to altered blood monocyte subpopulations. Monocytes were classified by flow cytometry as CD14^high^ including classical (CD14^high^CD16^-^) and intermediate (CD14^high^CD16^inter^) subpopulations, as well as CD14^low^ non-classical (CD14^low^CD16^high^) monocyte subpopulation, as previously described (**Supplementary Figure 1B**) (21). We found an increase in CD14^high^ macrophages, especially CD14^high^CD16^inter^ intermediate monocytes in obese subjects (**Figure 3C**). We extended these findings by correlating the monocyte subpopulations with gut inflammation and clinical data. Hereby, we found that the increase in intermediate monocytes correlated with the proportion of intestinal macrophage subpopulation P2 in the stomach and duodenum (**Figure 3D**). In sum, obese individuals had an increase in pro-inflammatory macrophage subpopulation P2 throughout the gastrointestinal tract. The concomitantly elevated intermediate blood monocytes suggested that the prevailing mechanism was enhanced by monocyte recruitment into the gut wall.

### Pro-Inflammatory Macrophage Subpopulation P2 and Intermediate Blood Monocytes Correlate with Body Weight Parameters

Next, we addressed the question whether gut inflammation proportionally increases with body weight. We therefore assessed different clinical parameters of body weight such as BMI, waist circumference, waist-to-hip-ratio (WHR), and waist-to-height ratio (WHtR) and correlated them with the frequencies of intestinal macrophage subpopulation P2 and intermediate blood monocytes. We found that the BMI had a positive relationship with the frequency of pro-inflammatory intestinal subpopulation P2 at all locations of the gastrointestinal tract tested (**Figure 3E**). Also other body weight parameters such as body weight, waist circumference, and waist-to-height ratio correlated with the frequency of P2 intestinal macrophages (**Supplementary Figures 4A–C**). They also positively correlated with the frequency of intermediate blood monocytes (**Figure 3E** and **Supplementary Figures 4A–C**, right panel). In contrast, the age or gender of the study participants did not correlate with an increase in the subpopulation P2 or intermediate monocytes (**Supplementary Figures 4D, E**). Thus, both intestinal macrophage subpopulation P2 and circulating intermediate monocytes showed a positive relationship with different clinical parameters of body weight.

### Pro-Inflammatory Macrophage Subpopulation P2 and Intermediate Blood Monocytes Correlate with Unhealthy Lifestyle Habits

**Figure 4:**
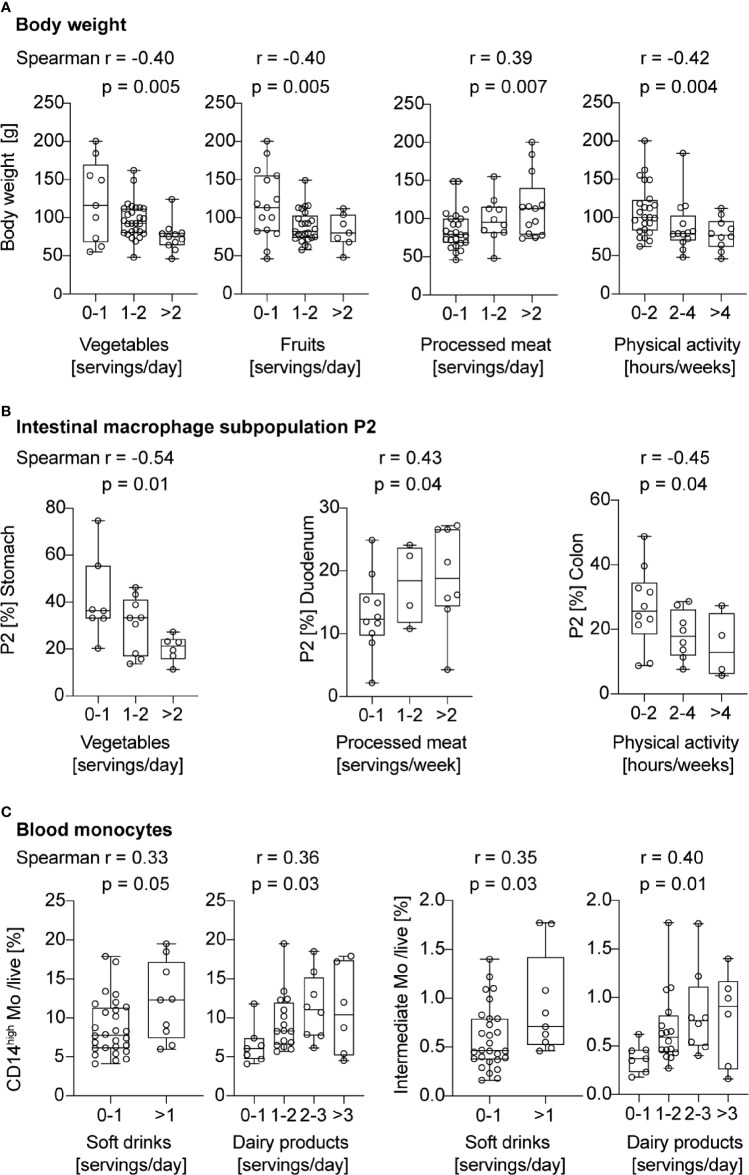
Pro-inflammatory macrophage subpopulation P2 and intermediate blood monocytes correlate with unhealthy lifestyle habits. **(A)** Spearman correlation between servings of vegetables, fruits, processed meat, or hours of physical activity and body weight. **(B)** Spearman correlation between servings of vegetables, processed meat, or hours of physical activity and the portion of intestinal macrophage subpopulation P2. **(C)** Spearman correlation between servings of soft drinks or dairy products, and CD14^high^ or intermediate blood monocytes. One data point represents one subject. For statistical analysis, Spearman correlation analysis was performed, and *p*-values < 0.05 were considered significant.

Previously, we found that mice had more pronounced gut inflammation when their HFD was lard-based rather than coconut-based (20). To assess whether human intestinal macrophages also respond to dietary cues, we evaluated dietary habits of the study participants by a nutrition and lifestyle questionnaire. The questionnaire was based on the Healthy Eating Index (23) and adapted to national dietary guidelines of Switzerland (**Table 2**). To assess the impact of specific food products and physical activity on gut inflammation and monocyte recruitment, different food groups or sports were correlated with pro-inflammatory intestinal macrophages P2 and intermediate monocytes. First, we confirmed the validity of the nutritional score as we found that higher body weight, expressed by different clinical body weight parameters (body weight, BMI, waist and hip circumference, WHtR), was associated with lower vegetable and fruit intake, higher processed meat consumption, less physical activity, and more soft drinks (**Figure 4A** and **Supplementary Figures 5A–D**). We found a higher frequency of pro-inflammatory macrophage subpopulation P2 in subjects with lower vegetable intake (stomach biopsies), with higher consumption of processed meat (duodenum biopsies), and with less physical activity (colon biopsies; **Figure 4B**). The other sections of the gastrointestinal tract did not show any correlation with different food components (**Supplementary Figures 5E–G**). Elevated frequencies of CD14^high^ blood monocytes and intermediate monocytes were found in patients with a higher intake of soft drinks and dairy products (**Figure 4C**). Thus, these data indicated that unhealthy lifestyle habits, such as sedentary lifestyle or unhealthy foods, correlated with gut inflammation as shown by increased pro-inflammatory macrophage subpopulation P2 and higher intermediate blood monocytes.

**Table 2:** Nutritional score with subgroups of dietary components.

**1. Vegetables**	Servings/day	>3 *(0.5)*	2-3 *(0.5)*	1-2 *(0.25)*	0-1 *(0)*	
	Type of vegetable - servings/week	≥ 4x green vegetables *(0.1)*	≥ 4x orange/red vegetables *(0.1)*	≥ 2x cabbage vegetables *(0.1)*	≥ 2x tuber vegetables *(0.1)*	≥ 1x legumes *(0.1)*
**2. Fruits**	Servings/day	> 3 *(1)*	2-3 *(1)*	1-2 *(0.5)*	0-1 *(0)*	
**3. Starch products**	Servings/day	> 4 *(0.5)*	3-4 *(1)*	1-2 *(0.5)*	0-1 *(0)*	
	Whole grain - servings/week	daily *(1)*	multiple times *(0.5)*	almost never *(0)*		
**4. Dairy products**	Servings/day	> 4 *(1)*	3-4 *(1.5)*	2-3 *(1)*	1-2 *(0.5)*	0-1 *(0)*
	Fat content	not fat reduced *(0)*	sometimes fat reduced *(0.25)*	mostly fat reduced *(0.5)*		
**5. Protein products (meat/eggs/meat substitutes)**	Servings/day	> 2 *(1)*	1-2 *(1.5)*	0.5-1 *(1)*	0-0.5 *(0)*	
	Processed meat - servings/week	> 2 *(0)*	1-2 *(0.1)*	0-1 *(0.25)*		
	Red meat - servings/week	> 3 *(0)*	0-3 *(0.25)*			
	For vegetarians/vegans - servings	> 3x/week legumes or daily nuts *(0.5)*	> 1x/week legumes or > 3x/week nuts *(0.25)*	0-1x/week legumes or 0-3x/week nuts *(0)*		
**6. Fat/oil**	Olive/rapeseed oil	mostly *(1)*	sometimes *(0.5)*	never *(0)*		
	Fish and nuts - servings/week	> 1x/week fish or	0-1x/week fish or			
		> 1x/day nuts *(1)*	0-1x/day nuts *(0)*			
**7. Salt**	Amount	a lot *(0)*	moderate *(0.5)*	minimum *(1)*		
	Fast food - times/week	> 2 *(0)*	0-2 *(0.5)*			
	Lunch in restaurant/canteen - times/week	> 2 *(0)*	0-2 *(0.5)*			
**8. Candies/snacks**	Servings/day	>4 *(0)*	2-4 *(1)*	0-2 *(2)*		
**9. Sweet beverages**	Servings/day	> 3 *(0)*	1-3 *(1)*	0-1 *(2)*		
**10. Alcohol**	Standard glasses/week - women	> 7 *(0)*	4-7 *(1)*	0-4 *(2)*		
	Standard glasses/week - men	> 14 *(0)*	8-14 *(1)*	0-8 *(2)*		
**11. Activity**	Weight dynamic last half year	Stable *(1)*	> 2kg weight gain *(0)*	> 4kg weight loss with overweight *(1)*	> 2kg loss with normal weight *(0)*	
	Daily activity during work	active* (0.5)*	sometimes active *(0.25)*	mostly sitting *(0)*		
	Sports - hours/week	> 4 *(0.5)*	2-4 *(0.25)*	0-2 *(0)*		
**Total score *(20)***						

Points per question are indicated in italic.

### Cardiovascular Risk Factors Other Than Obesity Do Not Affect Intestinal Macrophage Subpopulation P2 and Intermediate Blood Monocytes

**Figure 5:**
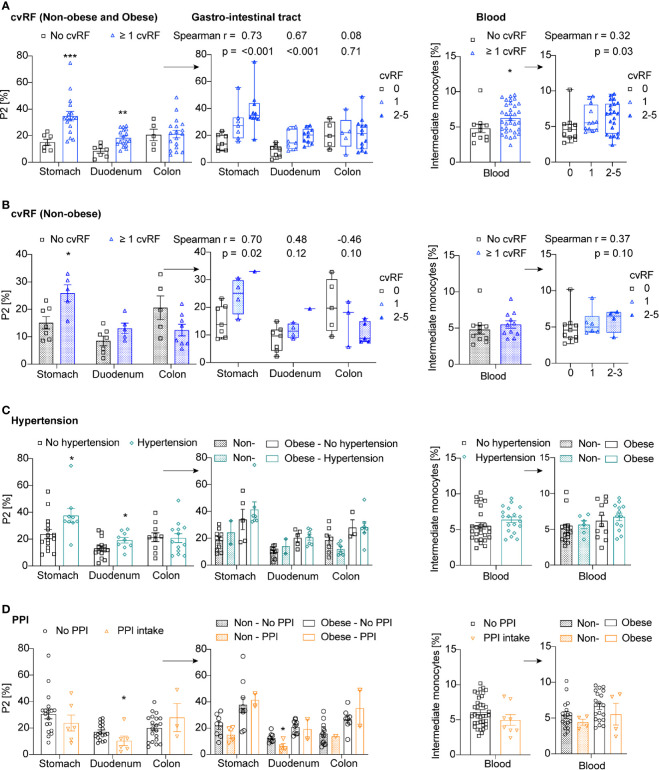
Cardiovascular risk factors other than obesity do not affect intestinal macrophage subpopulation P2 and intermediate blood monocytes. **(A–D)** Frequency of P2 intestinal macrophages in the stomach, duodenum, or colon transversum, and intermediate blood monocytes in patients with or without cardiovascular risk factors (cvRF) (blue) (**A**: Non-obese and obese patients, **B**: only non-obese patients), hypertension (green) **(C)**, and PPI intake (orange) **(D)**. The right panels are additionally stratified by the number of cvRFs (0, 1, >2) **(A, B)** or by the non-obese or obese body habitus **(C, D)**. One data point represents one subject. Statistical data are expressed as mean ± SEM. * *p* <0.05. ** *p* <0.01, *** *p* <0.001, unpaired Mann-Whitney U test with two tailed distribution. For Spearman correlation analysis, *p*-values < 0.05 were considered significant.

We next asked the question whether cardiovascular risk factors other than obesity affect intestinal macrophage and blood monocyte subpopulations. Of note, the risk factor smoking was not considered as active smokers were excluded beforehand. When first assessing a composite cardiovascular endpoint by including obesity, hypertension, dyslipidemia, diabetes, and family history for cardiovascular events (1 point for each factor), we found that the occurrence and number of cardiovascular risk factors positively correlated with intestinal macrophage subpopulation P2 in the stomach and the duodenum, as well as with the presence of intermediate blood monocytes (**Figure 5A**).

When assessing this composite cardiovascular endpoint in non-obese patients only, we found a positive correlation between the occurrence and number of cardiovascular risk factors and pro-inflammatory intestinal macrophage subpopulation P2 in the stomach; however, this was no longer significant in the duodenum or for intermediate blood monocytes (**Figure 5B**). As the patients with at least one cardiovascular risk factor had a significantly higher BMI than patients without cardiovascular risk factors (26.2kg/m^2^ versus 19.5kg/m^2^, p-value <0.005), this correlation could still be driven by body weight differences. To exclude residual confounding by body weight, we analyzed each cardiovascular risk factor separately and stratified the analysis by the non-obese or obese state of the study participants. Subjects with hypertension had more pro-inflammatory intestinal macrophages P2 in the stomach; however, this effect was no longer detectable when patients were stratified by body weight (**Figure 5C**). The presence of dyslipidemia or diabetes did not seem to affect the innate immune response in the gut or blood, however, only few overtly diabetic subjects were included in the study (**Supplementary Figures 6A, B**). Analyzing the data by multiple linear regression adjusted for BMI revealed the same results, namely that presence of cardiovascular risk factors other than obesity did not impact on the frequency of P2 macrophages (data not shown).

To support these findings, we measured C-reactive protein (CRP), liver enzymes, and plasma lipids at the time of biopsy. We correlated these laboratory parameters with the innate immune response in the gut and blood. CRP as a measure for low-grade systemic inflammation in obesity (24) showed a trend to a positive correlation with pro-inflammatory intestinal macrophage subpopulation P2 in the stomach (p=0.08) and the duodenum (p=0.06) (**Supplementary Figure 6C**). In the colon and the blood, no relationship was found between CRP and the innate immune response. Next, we assessed lipid levels, such as total cholesterol, triglycerides, low-density lipoprotein (LDL) and high-density lipoprotein (HDL), in relation to the gut and systemic innate immune response. These lipid parameters did not correlate with the innate immune response, with the exception of a negative correlation between HDL and pro-inflammatory intestinal macrophage subpopulation P2 in the duodenum (**Supplementary Figure 6D**). Also there was no correlation between the liver enzyme alanine transaminase (ALAT) and the frequency of intestinal macrophage P2 population or blood monocytes observed (**Supplementary Figure 6E**). Hence, we found that obesity was the main cardiovascular risk factor driving to increase in inflammatory intestinal macrophages and intermediate monocytes.

### Preliminary Data Show Reduced Intestinal Macrophage Subpopulation P2 in the Duodenum of Patients Using Proton Pump Inhibitors (PPIs)

To assess a potential impact of drugs on the innate immune response we observed, we correlated the intake of drugs with pro-inflammatory intestinal macrophage subpopulation P2 and blood monocytes. Study participants on immunosuppressive drugs, steroids, or non-steroidal antirheumatic drugs were excluded from the study. Interestingly, proton pump inhibitor (PPI) intake correlated with reduced frequencies of intestinal P2 macrophages in the duodenum (**Figure 5D**). This reduction of pro-inflammatory intestinal macrophages was evident independent of the body weight. In the stomach and colon, however, no association was found between PPI intake and either intestinal macrophage subpopulation P2 or intermediate blood monocytes. Other drugs were not correlated with intestinal macrophages or blood monocytes (not shown), including drugs such as aspirin, statins, antihypertensive drugs, antidiabetic drugs, levothyroxine, calcium, vitamin D, hormone replacement therapy, and antipsychotic/antidepressant/antiepileptic drugs. Hence, preliminary data indicated a reduced intestinal macrophage subpopulation P2 in the duodenum of patients taking proton pump inhibitors (PPIs).

## Discussion

Due to the continuous exposure to antigens from foods, microbiota and metabolites, the gut could be the organ where inflammation origins in metabolic disease. Previously, an increase in the number of leukocytes in the intestinal mucosa and a change toward pro-inflammatory cell types like Th1 cells, total macrophages, dendritic cells, and NK cells has been found in obese humans (25, 26). However, a detailed characterization of intestinal macrophage subpopulations in human obesity was missing so far. Our study represents a comprehensive analysis of monocyte and intestinal macrophage subpopulations in healthy and obese individuals.

In the healthy gut, the number of macrophages is most densely distributed in the colorectal mucosa (27, 28). We extended these findings by discriminating five distinct macrophage subpopulations (monocyte-derived P1, P2, intermediate stage P3 and resident/mature P4, P5). This gating strategy is based on Bain et al. (5), but also corresponds to other published gating strategies of human intestinal macrophages as outlined in in **Supplementary Table 3**. Based on the literature, monocyte-derived P1, P2 are considered as pro-inflammatory as they are increased during inflammatory processes in the gut and able to secrete pro-inflammatory cytokines (5, 28, 29). In contrast, resident/mature P4, P5 intestinal macrophages have been shown to produce IL-10 (28). We found that anti-inflammatory/resident macrophages (subpopulation P5) increase along the gastrointestinal tract, while the monocyte-derived, pro-inflammatory and intermediate subpopulations (P1-P3) decrease. This was observed in both non-obese and obese individuals. The number of intestinal macrophages thus closely follows the density and diversity of gut microbiota (30). An interaction between intestinal macrophages and gut microbiota is supported by the fact that germ-free mice have markedly reduced immune cells in the gut wall (3). This suggests that gut microbiota are driving monocyte recruitment to the gut wall, and thereby determine the presence of intestinal macrophages.

Based on our preclinical data in mice fed HFD (20), we compared intestinal macrophage subpopulations in non-obese and obese individuals and found a distinct increase in the pro-inflammatory subpopulation P2 in obese subjects in all sections of the gastrointestinal tract analyzed. Our findings are reminiscent of increased pro-inflammatory intestinal macrophages described in infectious and inflammatory bowel diseases (1, 5, 28, 31), where newly recruited monocytes seem to be arrested in an immature state without further differentiating into anti-inflammatory/resident macrophages (5). Whether increased monocyte-derived, pro-inflammatory intestinal macrophages in obese subjects directly induce systemic inflammation and/or glucose tolerance (i.e. *via* pro-inflammatory cytokines) needs to be addressed in future studies. The increase in inflammatory intestinal macrophages could occur either in response to an altered microenvironment in the gut or already prior to that in the bone marrow. A changed microenvironment in the gut could be due to inflammatory factors or a loss of intrinsic maturation cues, which would then impact on the normal differentiation process of intestinal macrophages. For example, it has been suggested that intrinsic IFN-gamma signaling in the gut is important for maintaining the inflammatory macrophage phenotype in IBD, potentially *via* epigenetic regulation (31, 32). Alternatively, myeloid precursors or monocytes replenishing intestinal macrophages could already be altered in the bone marrow. Previously, activation of bone marrow myeloid cells prior to their recruitment to the gut has been described in gastrointestinal infections and colitis in mice (33, 34). Additionally, HFD has been shown to alter myeloid progenitor cells in the bone marrow by NLRP3-dependent epigenomic and transcriptomic reprogramming (35). While characterization of myeloid cells in the bone marrow was beyond the scope of our study, we found an increase in intermediate blood monocytes in obese subjects consistent with previous findings, but no increase in non-classical monocytes (36, 37). Intermediate monocytes are known to exhibit a pro-inflammatory profile (38), which is further enhanced in obesity as shown by an increased migratory capacity and response upon TLR-stimulation (39). Thus, activation of myeloid cells in obesity could originate either in the gut or the bone marrow before replenishment of intestinal macrophages.

Currently, the signals that instruct such education of myeloid cells have not been identified. Our data links unhealthy lifestyle behaviors (i.e. low vegetable intake or high consumption of processed meat) to changes in gut innate immunity and increased intermediate monocytes. Previous studies have also established a link between nutrients and intestinal macrophage function or differentiation. For example, amino acids have been shown to regulate intestinal macrophage function (40, 41). Furthermore, a loss of branched-chain amino acid transporter CD98hc impedes normal differentiation of intestinal macrophages towards an anti-inflammatory state (42). As changes in gut microbiota have been well established in obesity, also gut microbiota and their metabolites could mediate gut inflammation in metabolic disease (43). One potential explanation linking diet, gut microbiota and intestinal macrophages in obesity could be *via* metabolite-sensing G protein-coupled receptors (GPCRs). GPCRs sense metabolites of gut microbial fermentation from fibers such as short chain fatty acids, which have been suggested to exert beneficial effects on experimental colitis in mice (44). However, it remains to be determined whether changes in gut microbiota and increased inflammatory macrophages in the gut of obese individuals are causally linked or a concurrent phenomena. Importantly, the increase in pro-inflammatory intestinal macrophages we observed was present in all sections of the gastrointestinal tract, including stomach, duodenum and colon. This argues against a purely microbiota-mediated mechanism of gut inflammation in obesity as the stomach and the colon harbor vastly different microbiota (22).

The question remains whether attenuation of gut inflammation could potentially lead to reduced systemic inflammation and improved metabolic health. Immune-modulatory treatments directing the differentiation of intestinal macrophages towards an anti-inflammatory/resident phenotype have mostly been studied in the context of IBD. For example, anti-TNF−alpha antibodies have been shown to decrease pro-inflammatory (45) and increase anti-inflammatory intestinal macrophage subpopulations in IBD, potentially contributing to disease resolution (46, 47). Also, classical immunosuppressive drugs used in IBD such as 5-aminosalicylic acid (5-ASA) have been described to dampen pro-inflammatory macrophages by inhibiting NF-kappaB activation (48). Interestingly, mice fed HFD incorporated with 5-ASA treatment had improved glucose tolerance and insulin sensitivity along with reduced gut and adipose tissue inflammation (25). To directly target intestinal macrophages, Kawano and colleagues generated an intestinal epithelial cell-specific Ccl2 knockout mouse model (17). They showed that decreased colonic infiltration of pro-inflammatory macrophages in these mice led to an improved glucose tolerance and insulin sensitivity (17). Complementary to this work, we recently were able to pharmacologically deplete macrophages in the colon by intrarectal clodronate liposome injections in mice, which resulted in improved glycemic control (20). In the current study, the finding that P2 macrophages in the duodenum were reduced in non-obese subjects taking PPIs have yet to be interpreted with caution as prospective studies are needed. Also, it is unclear whether attenuation of gut inflammation only in the duodenum is sufficient to bring about beneficial effects on inflammation and glucose homeostasis. Besides pharmacological interventions, weight loss might also impact gut inflammation. For example, one study showed that diet-induced weight loss in obese individuals reduced colorectal inflammation as shown by a decrease in CD163^+^ macrophages and modulation of inflammatory gene pathways in the gut (49). Thus, both pharmacological interventions and weight loss could have the potential to beneficially affect gut inflammation and thereby metabolic outcomes.

Due to the limited amount of tissue collected during the endoscopies and the distinction of several macrophage subpopulations already involving a multicolor flow cytometry panel, it was not possible to simultaneously assess other immune cell subpopulations that could have potentially been altered in an obese state. However, it is likely that there is a close interaction between various immune cell compartments and also non-immune cells present in the gastrointestinal wall. The gene expression analysis from whole duodenum tissue of obese and non-obese subjects can be regarded as an integrative product of the different cell types residing in the gastrointestinal wall. To further link the observed increase in pro-inflammatory macrophage subpopulations with gene expression changes, a single-cell approach will be needed in future studies.

In sum, our study establishes a role of mucosal innate immunity in obese humans. We found a distinct increase in the pro-inflammatory intestinal macrophage subpopulation P2 in obese subjects, reminiscent of the inflammatory shift reported in IBD. This innate response seems to be triggered by unhealthy lifestyle behaviors, such as low vegetable intake, high consumption of processed meat or physical inactivity. Future studies will need to identify the site of innate immune cell activation and the signals triggering this response. Understanding the role of mucosal innate immunity in human obesity might open up new possibilities for immune-modulatory treatments in metabolic disease.

## Data Availability Statement

The original contributions presented in the study are included in the article/**Supplementary Material**. Further inquiries can be directed to the corresponding author.

## Ethics Statement

The studies involving human participants were reviewed and approved by Ethikkommission Nordwest- und Zentralschweiz. The patients/participants provided their written informed consent to participate in this study.

## Author Contributions

TR, RF, RM, CC-W were involved with study concept and design. Patient identification/recruitment and obtainment of clinical data were performed by RF, RM, and CC-W. Surgical and biopsy specimen were obtained by RS, IL, JP, JN, TD, and PH. Multi-color flow cytometry method validation and data analysis was done by TR with the support of DL. TR, LK, and ZB performed experimental procedures and T.R., L.K., data acquisition. TR, LK, RF, and RM were involved in data interpretation and statistical analysis. TR and CC-W obtained the funds to perform this work. The figures were created by TR and tables by TR, RF, RM. The manuscript was drafted by TR, RF, RM, CC-W. CC-W is the guarantor of this work. All authors contributed to the article and approved the submitted version.

## Funding

This study was supported by grants from the Swiss National Science Foundation (PZ00P3_161135), the Goldschmidt-Jacobson Foundation, the Jubiläumsstiftung Swiss Life, the Olga Mayenfisch Foundation, the Foundation Basler Diabetesgesellschaft (to CCW), the Nikolaus and Bertha Burckhardt-Bürgin-Stiftung, and from the Research Fund for Excellent Junior Researchers of the University of Basel (to TR).

## Conflict of Interest

The authors declare that the research was conducted in the absence of any commercial or financial relationships that could be construed as a potential conflict of interest.
