# Supplementary material for: Obesity in Humans Is Characterized by Gut Inflammation as Shown by Pro-Inflammatory Intestinal Macrophage Accumulation
Source: Front Immunol. 2021 May 12;12:668654. doi: 10.3389/fimmu.2021.668654 (PMC8158297; doi:10.3389/fimmu.2021.668654)
Supplement: Supplementary file 1 [file DataSheet_1.docx]

Supplementary Figures and Titles

**Supplementary Figure 1**

**
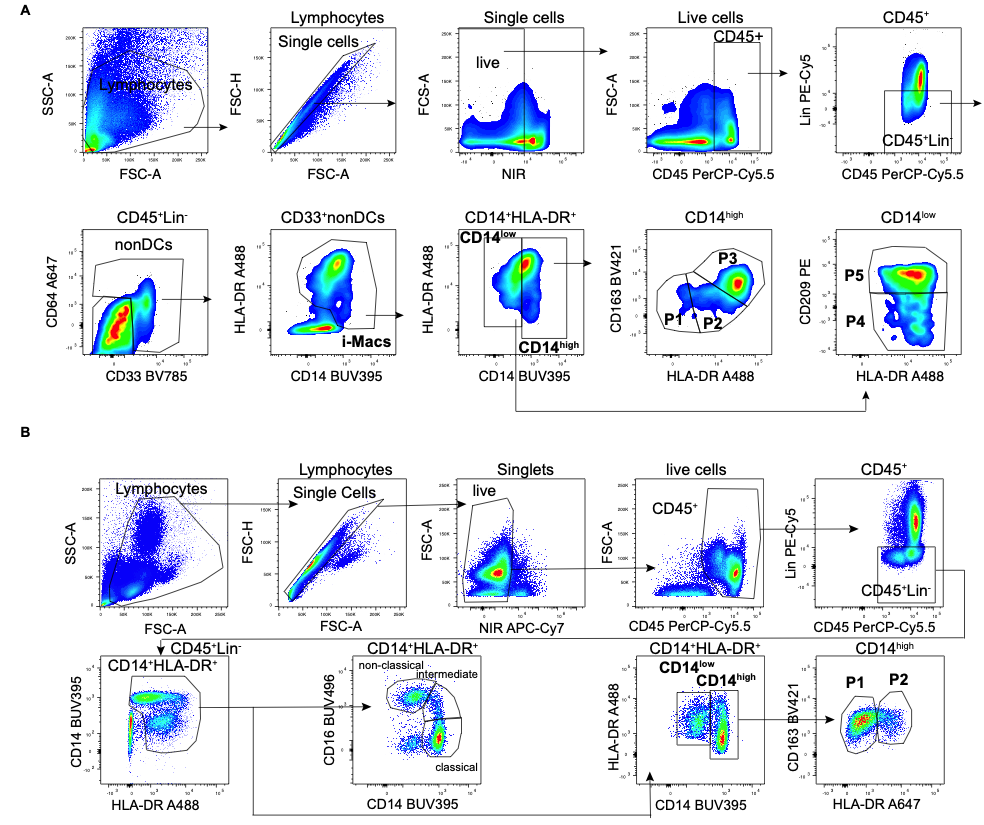
**

**Supplementary Figure 1: Flow cytometry gating strategy to identify human intestinal macrophage and blood monocyte subpopulations.** **(A)** Intestinal Macrophages were identified as live CD45^+^Lin^-^(CD19^-^CD3^-^CD56^-^CD20^-^TCRαβ^-^) CD33^+^nonDCs (CD33^-^CD64^-^) and CD14^low to high^HLA-DR^low to high^. Monocyte-derived were characterized as CD14^high^ (P1, P2, P3) and resident macrophages as CD14^low^ (P4, P5). CD14^high^ macrophages include pro-inflammatory P1(CD163^low^HLA-DR^low^), P2 (CD163^low^HLA-DR^inter^) and intermediate P3 (CD163^high^HLA-DR^high^) subpopulations whereas CD14^low^ macrophages harbors the anti-inflammatory/ resident P4 (CD209^low^HLA-DR^inter^), and P5 (CD209^high^HLA-DR^high^) subpopulations. **(B)** Blood monocytes were classified as CD14^high^CD16^-^ classical, CD14^high^CD16^inter^ intermediate, and CD14^low^CD16^high^ non-classical monocytes as well as CD14^high^ (P1, P2) blood monocytes.

**Supplementary Figure 2
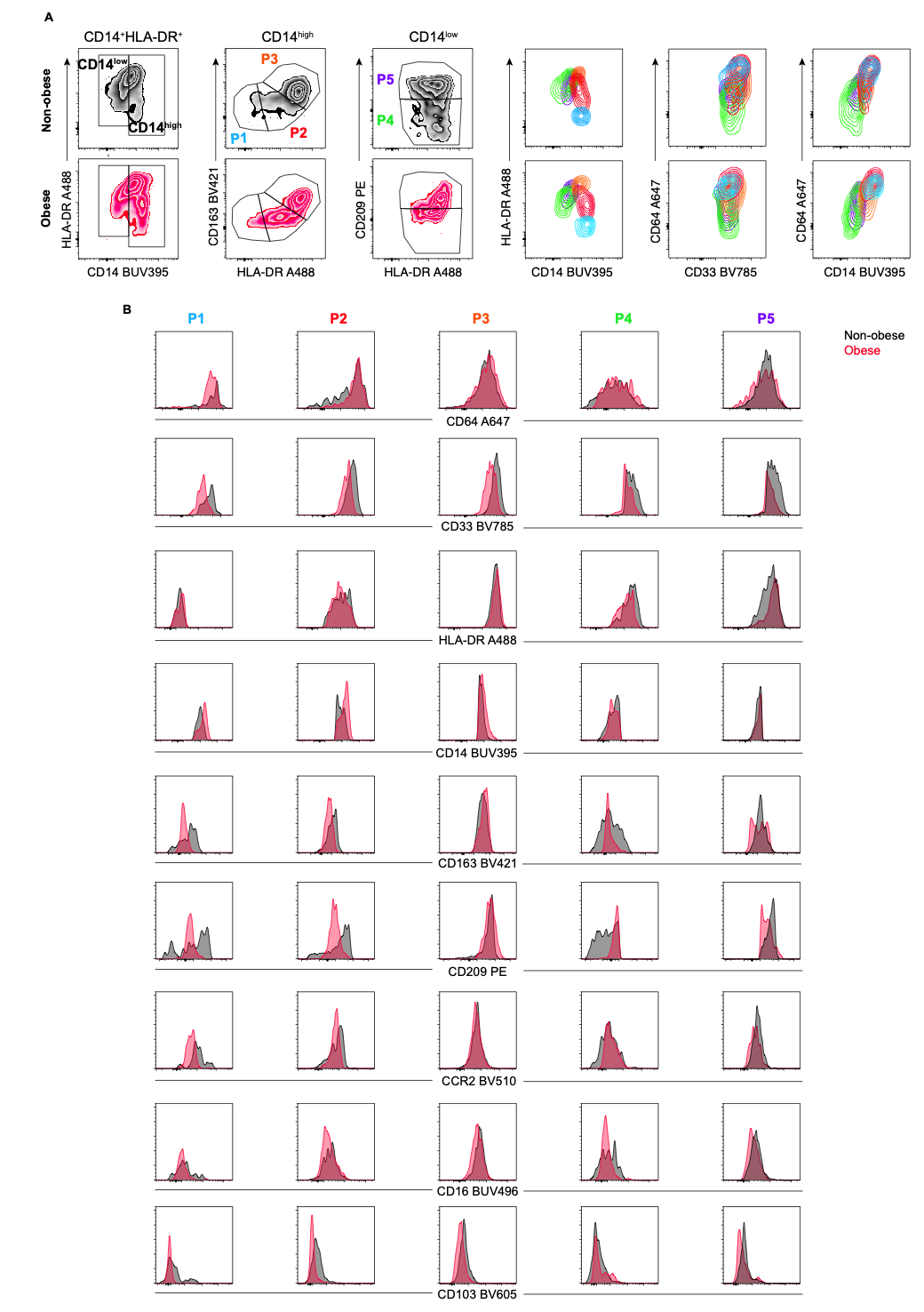
**

**Supplementary Figure 2: Marker characteristics of the different human intestinal macrophage subpopulations (P1-P5).** **(A)** Representative flow cytometric analysis of CD14^high^ P1-P3 and CD14^low^ P4, P5 intestinal macrophage subpopulations and their expression pattern of CD14 vs HLA-DR, CD33 vs CD64 and CD14 vs CD64. **(B)** Comparison of the histograms of different surface markers within the defined P1-P5 intestinal macrophages in a representative duodenum biopsy of a non-obese (black) and obese (red) subject.

**Supplementary Figure 3**

**
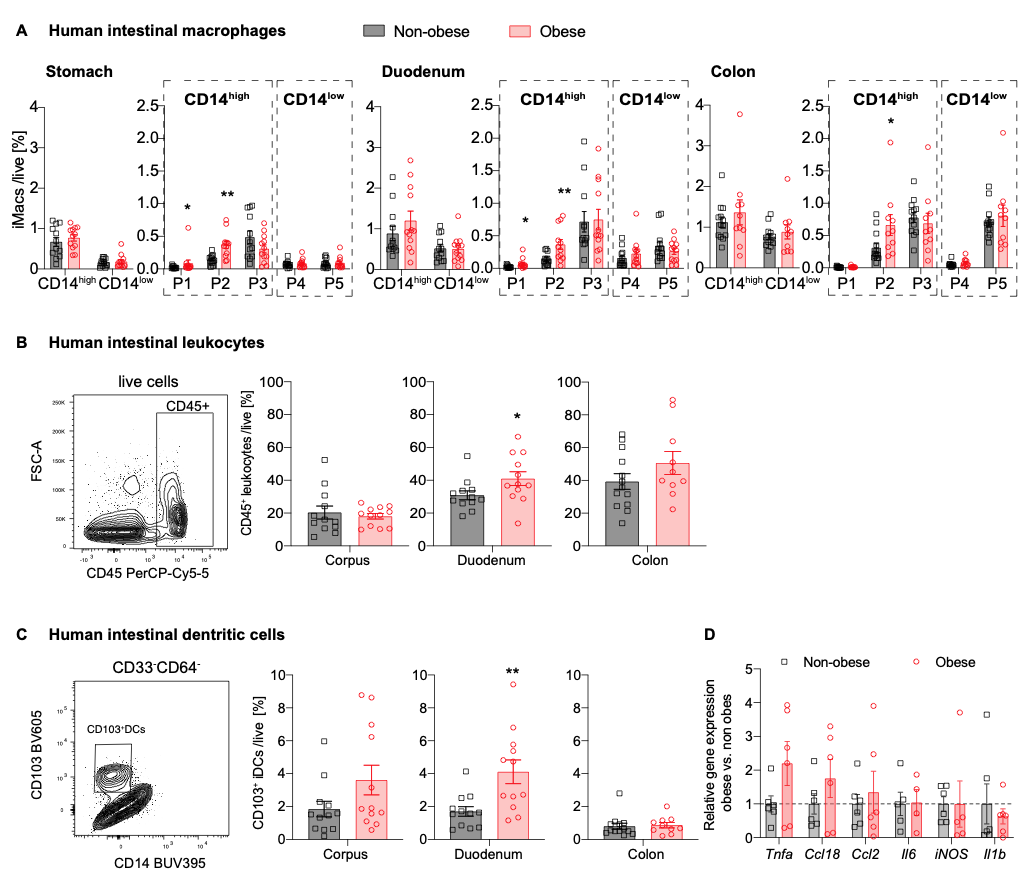
**

**Supplementary Figure 3: Intestinal leukocytes, dendritic cells, macrophage subpopulations and blood monocytes in obesity. (A-C)** Frequency of human intestinal macrophage subpopulations **(A)**, CD45^+^ leukocytes **(B)** and CD33^-^CD64^-^CD103^+^ dendritic cells (DCs) **(C)** of live cells (non-obese n=12-13, obese n=10-12 subjects). **(D)** Relative gene expression of pro-inflammatory genes in duodenum tissue of obese (red) subjects compared to non-obese (black). One data point represents one subject. Statistical data are expressed as mean±SEM. **p*<0.05. ***p*<0.01, ****p*<0.001, unpaired Mann-Whitney U test with two tailed distribution.

**Supplementary Figure 4**

**Supplementary Figure 4: Intestinal macrophage subpopulation P2 and intermediate blood monocytes correlate with body weight parameters but are independent of age or gender. (A-D)** Spearman correlation of intestinal macrophage subpopulation P2 of stomach, duodenum, and colon transversum or intermediate blood monocytes with body weight parameters or age: body weight **(A)**, waist circumference **(B)**, waist-to-height ratio (WHtR) **(C)** and age **(D)**. **(E)** Percentage of intestinal macrophage subpopulation P2 of stomach, duodenum, or colon, and intermediate blood monocytes of male (black) and female (red) individuals. One data point represents one subject. For Spearman correlation analysis, values above Mean+2SD were excluded **(A-D)**, and *p*-values < 0.05 were considered significant.

**Supplementary Figure 5**

**Supplementary Figure 5: Eating behavior and physical activity correlate with body weight parameters and modulate intestinal inflammation as well as blood monocyte recruitment. (A-D)** Spearman correlation of nutritional scores and BMI **(A)**, waist circumference **(B)**, hip circumference **(C)**, and wait-to-height-ratio (WHtR) **(D)**. **(E-G)** Spearman correlation of intestinal macrophage subpopulation P2 and servings of vegetables **(E)**, processed meat **(F)**, or hours of physical activity **(G)**. For Spearman correlation analysis, *p*-values < 0.05 were considered significant.

**Supplementary Figure 6**

**Supplementary Figure 6: The influence of cardiovascular risk factors and lipids on intestinal macrophage subpopulation P2 and intermediate blood monocytes. (A/B)** Frequency of P2 intestinal macrophages in the stomach, duodenum, or colon transversum, and intermediate blood monocytes in subjects with or without diabetes **(A)** or dyslipidemia **(B)**. The right panels are additionally stratified by the non-obese or obese body status. **(C-E)** Spearman correlation between the intestinal macrophage subpopulation P2 of stomach, duodenum, or colon transversum, or intermediate blood monocytes with C-reactive protein (CRP) **(C)**, alanine amino transferase (ALAT) **(D)**, and high-density lipoprotein (HDL) **(E)**. For Spearman correlation analysis, values above Mean+2SD were excluded **(A-B)**, and *p*-values < 0.05 were considered significant.

Supplementary Table 1

| **REAGENT or RESOURCE** | **SOURCE** | **IDENTIFIER** |
| --- | --- | --- |
| Anti-human CD19 (HIB19) PE-Cy5 | Biolegend | Cat#302209; RRID: [AB_314239](http://antibodyregistry.org/AB_314239) |
| Anti-human CD3 (UCHT1) PE-Cy5 | Biolegend | Cat#300410; RRID: [AB_314064](http://antibodyregistry.org/AB_314064) |
| Anti-human CD56 (5.1H11) PE-Cy5 | Biolegend | Cat#362515; RRID: [AB_2564088](http://antibodyregistry.org/AB_2564088) |
| Anti-human CD20 (2H7) PE-Cy5 | Biolegend | Cat#302307; RRID: [AB_314255](http://antibodyregistry.org/AB_314255) |
| Anti-human TCRαβ (IP26) PE-Cy5 | Biolegend | Cat#306710; RRID: [AB_314648](http://antibodyregistry.org/AB_314648) |
| Anti-human CD45 (HI30) PerCP-Cy5.5 | Biolegend | Cat#304027; RRID: [AB_1236444](http://antibodyregistry.org/AB_1236444) |
| Anti-human CD64 (10.1) A647 | Biolegend | Cat#305012; RRID: [AB_528867](http://antibodyregistry.org/AB_528867) |
| Anti-human HLA-DR (L243) A488 | Biolegend | Cat#307619; RRID: [AB_493176](http://antibodyregistry.org/AB_493176) |
| Anti-human CD14 (MφP9) BUV395 | BD Biosciences | Cat#563562; RRID: [AB_2744288](http://antibodyregistry.org/AB_2744288) |
| Anti-human CD16 (3G8) BUV496 | BD Biosciences | Cat#564654; RRID: [AB_2744294](http://antibodyregistry.org/AB_2744294) |
| Anti-human CD209 (9E9A) PE | Biolegend | Cat#330105; RRID: [AB_1134060](http://antibodyregistry.org/AB_1134060) |
| Anti-human CD163 (GH1/61) BV421 | Biolegend | Cat#333611; RRID: [AB_2562462](http://antibodyregistry.org/AB_2562462) |
| Anti-human CCR2 (KO36C2) BV510 | Biolegend | Cat#357217; RRID: [AB_2566503](http://antibodyregistry.org/AB_2566503) |
| Anti-human CD33 (WM53) BV785 | Biolegend | Cat#303427; RRID: [AB_2650887](http://antibodyregistry.org/AB_2650887) |
| Anti-human CD103 (Ber-ACT8) BV605  Anti-human CD11b BV711  Anti-human CD11c PE-Dazzle | Biolegend  Biolegend  Biolegend | Cat#350217 ;RRID: [AB_2564282](http://antibodyregistry.org/AB_2564282)  Cat#301344;RRID: [AB_2563792](http://antibodyregistry.org/AB_2563792)  Cat#301641; RRID: [AB_2564082](http://antibodyregistry.org/AB_2564082) |
| **Biological Samples** | | |
| Human colon biopsies | This paper | #1CUSB,#2CUSB, #10CUSB, #14CUSB, #17CUSB, #19CBDA, #20CUSB, #21CMDP,#22CMDP, #27CUSB, #28CUSB, #29CUSB, #31CMDP, #32CUSB, #34CMDP, #37CMDP, #38CMDP, #47CMDP, #60CMDP, #66CMDP, #68G(B)USB, #69CMDP, #74CMDP |
| Human stomach and duodenum biopsies | This paper | #3GUSB, #5GUSB, #6GUSB, #7GUSB, #11GUSB, #12GUSB, #26GUSB, #30GUSB, #36GBMDP, #40GMDP, #41GBUSB, #42G(B)MDP, #44GBMDP/USB, #45GMDP, #46GMDP, #56GUSB, #57GMDP, #59GBMDP, #61GMDP, #67GMDP, #68G(B)USB, #71GBUSB, #72GUSB, #79GMDP |
| Human Blood | This paper | #5GUSB, #6GUSB, #7GUSB, #10CUSB, #11GUSB, #12GUSB, #14CUSB, #17CUSB, #18GBDA, #19CBDA, #20CUSB, #26GUSB, #27CUSB, #28CUSB, #29CUSB, #30GUSB #31CMDP, #32CUSB, #34CMDP, #36GBMDP, #37CMDP, #38CMDP, #41GBUSB, #42G(B)MDP, #44GBMDP/USB, #45GMDP, #46GMDP, #47CMDP, #52BUSB, #56GUSB, #57GMDP, #59GBMDP, #60CMDP, #66CMDP, #67GMDP, #68G(B)USB, #69CMDP, #70(G)BUSB, #71GBUSB, #72GUSB, #74CMDP, #79GMDP |
| **Chemicals, Peptides, and Recombinant Proteins** | | |
| Collagenase VIII | Sigma-Aldrich | Cat#C2139 |
| DNAse I | Roche | Cat#11284932001 |
| Percoll | GE Healthcare | Cat#GE17-0891-01 |
| Zombie NIR Fixable Viability Kit | Biolegend | Cat#423105 |
| **Software and Algorithms** | | |
| Prism8 | GraphPad Software, LLc. | <https://www.graphpad.com> |
| FlowJo (version 10.61) | Becton Dickinson & Company (BD) | <https://www.flowjo.com/solutions/flowjo/downloads> |

**Supplementary Table 1: Key resources**

| Supplementary Table 2: Baseline characteristics of study participants in different intervention groups (gastric, colonic, blood or bariatric samples) | | | | | | | | | | |
| --- | --- | --- | --- | --- | --- | --- | --- | --- | --- | --- |
|  | **Biopsy of gastroscopy** | | | **Biopsy of colonoscopy** | | | **Blood samples** | | | **Bariatric samples** |
| Characteristic | **Non-obese (n=12)** | **Obese (n=12)** | **p-value** | **Non-obese (n=13)** | **Obese (n=10)** | **p-value** | **Non-obese (n=21)** | **Obese (n=24)** | **p-value** | **Obese (n=6)** |
| Age (years) | 52.3±4.9 | 45.4±4.8 | 0.38 | 58.9±4.0 | 60.9±4.8 | 0.75 | 51.9±3.6 | 50.8±3.6 | 0.88 | 42. 3±4.8 |
| Male (%) | 7 (58) | 8 (67) | 1.00 | 5 (38) | 4 (40) | 1.00 | 9 (43) | 13 (54) | 0.55 | 4 (67) |
| Female (%) | 5 (42) | 4 (33) |  | 8 (62) | 6 (60) |  | 12 (57) | 11 (46) |  | 2 (33) |
| Caucasian (%) | 11 (92) | 12 (100) | 1.00 | 12 (92) | 9 (90) | 1.00 | 18 (86) | 23 (96) | 0.33 | 5 (83) |
| Other (%) | 1 (8) | 0 (0) |  | 1 (8) | 1 (10) |  | 3 (14) | 1 (4) |  | 1 (17) |
| BMI (kg/m^2^) | 22.3±1.2 | 40.6±2.3 | <0.001 | 24.3±0.8 | 36.3±1.1 | <0.001 | 23.5±0.7 | 38.9±1.3 | <0.001 | 44.4±3.0 |
| Weight (kg) | 68.4±4.1 | 131.6±10.1 | <0.001 | 69.6±2.8 | 102.7±5.9 | <0.001 | 69.5±2.4 | 119.9±6.3 | <0.001 | 144.0±13.7 |
| Waist (cm) | 89.6±3.0 | 131.1±4.9 | <0.001 | 90.4±4.0 | 124.5±5.2 | <0.001 | 89.5±2.6 | 126.5±3.6 | <0.001 | 137.6±8.2 |
| WHR | 0.98±0.02 | 1.04±0.02 | 0.03 | 0.95±0.02 | 1.01±0.02 | 0.04 | 0.96±0.01 | 1.01±0.02 | 0.01 | 1.00±0.04 |
| WHtR | 0.52±0.01 | 0.71±0.03 | <0.001 | 0.55±0.02 | 0.73±0.02 | <0.001 | 0.54±0.01 | 0.72±0.02 | <0.001 | 0.78±0.04 |
| Diabetes (%) | 0 (0) | 3 (25) | 0.22 | 0 (0) | 2 (20) | 0.18 | 0 (0) | 5 (21) | 0.05 | 2 (33) |
| Obesity (%) | 0 (0) | 12 (100) | <0.001 | 0 (0) | 10 (100) | <0.001 | 0 (0) | 24 (100) | <0.001 | 6 (100) |
| Hypertension (%) | 2 (17) | 7 (58) | 0.09 | 6 (46) | 7 (70) | 0.40 | 6 (29) | 13 (54) | 0.13 | 2 (33) |
| Dyslipidemia (%) | 3 (25) | 2 (17) | 1.00 | 6 (46) | 5 (50) | 1.00 | 6 (29) | 7 (29) | 1.00 | 3 (50) |
| Family history (%) | 1 (8) | 1 (8) | 1.00 | 2 (15) | 1 (10) | 1.00 | 3 (14) | 1 (4) | 0.33 | 0 (0) |
| CRP (mg/l) | 1.8±0.5 | 7.9±1.9 | 0.01 | 1.3±0.3 | 4.8±1.8 | 0.05 | 1.6±0.3 | 6.3±1.2 | <0.001 | 4.6±1.3 |
| ASAT (U/l) | 23.2±2.1 | 25.8±2.3 | 0.45 | 23.8±4.6 | 23.8±1.6 | 0.55 | 23.6±2.2 | 24.5±1.3 | 0.43 | 25.0±1.9 |
| ALAT (U/l) | 23.4±3.0 | 38.4±4.6 | 0.03 | 27.7±5.0 | 37.7±7.1 | 0.35 | 26.2±2.8 | 37.3±3.5 | 0.02 | 34.0±4.8 |
| Chol. (mmol/l) | 4.9±0.3 | 4.8±0.3 | 0.82 | 4.6±0.4 | 4.9±0.4 | 0.50 | 4.7±0.2 | 4.8±0.2 | 0.80 | 4.5±0.3 |
| TG (mmol/l) | 1.2±0.3 | 1.5±0.2 | 0.25 | 1.0±0.2 | 1.9±0.4 | 0.02 | 1.2±0.2 | 1.6±0.2 | 0.02 | 1.7±0.3 |
| LDL (mmol/l) | 2.5±0.3 | 2.9±0.2 | 0.35 | 2.6±0.3 | 2.6±0.3 | 0.84 | 2.6±0.2 | 2.8±0.2 | 0.43 | 2.8±0.2 |
| HDL (mmol/l) | 1.8±0.2 | 1.2±0.1 | <0.004 | 1.5±0.1 | 1.5±0.2 | 0.41 | 1.6±0.1 | 1.3±0.1 | 0.01 | 1.0±0.1 |

**Supplementary Table 2: Baseline characteristics of study participants in different intervention groups (gastric, colonic, blood or bariatric samples).** Data were expressed as mean ± standard error of the mean (SEM) and number (%). Waist-to-Hip Ratio (WHR) was calculated as waist circumference divided by hip circumference, and Waist-to-Height Ratio (WHtR) was calculated as waist circumference divided by height. BMI: Body mass index; WHR: Waist-to-Hip Ratio; WHtR: Waist-to-Height Ratio; cvRF: cardiovascular risk factors; CRP: C-reactive protein; ASAT: Aspartate Aminotransferase; ALAT: Alanine Aminotransferase; Chol.: cholesterol; LDL: Low-density lipoprotein; HDL: High-density lipoprotein; TG: triglycerides.

| **Supplementary Table 3. Comparison of different gating strategies of human intestinal macrophage subpopulations** | | | |
| --- | --- | --- | --- |
| **Our gating strategy** | **Bain et al.** | **Bujko et al.** | **Bernardo et al.** |
| P1 (CD14^high^HLA-DR^low^CD163^low^) | P1 (CD14^high^HLA-DR^low^CD163^low^CD209^low^CD11c^high^) | Mf1 (CD14^high^HLA-DR^inter^CD11c^high^) | (CD64^+^CD14^+^CD11c^high^) |
| P2 (CD14^high^HLA-DR^inter^CD163^low^) | P2 (CD14^high^HLA-DR^high^CD163^low^CD209^low^CD11c^high^) | Mf1 (CD14^high^HLA-DR^inter^CD11c^high^) | CD64^+^CD14^+^CD11c^high^ |
| P3 (CD14^high^HLA-DR^high^CD163^high^) | P3 (CD14^high^HLA-DR^high^CD163^high^CD209^high^CD11c^low^) | (Mf3 (CD14^+^HLA-DR^high^CD11c^-^)) | CD64^+^CD14^+^CD11c^dim^ |
| P4 (CD14^low^HLA-DR^high^CD209^low^) | P4 (CD14^low^HLA-DR^high^CD209^low^CD163^low^CD11c^inter^) | Mf2 (CD14^low^HLA-DR^high^CD11c^+^) | CD64^+^CD14^+^CD11c^dim^ |
| P5 (CD14^low^HLA-DR^high^CD209^high^)  Other Notes: | P5 (CD14^low^HLA-DR^high^CD209^high^CD163^high^CD11c^-^)  (CD14^high^ (P1-P3)= CXCR1^inter^, P4 = later transition of CXCR1^inter^, P5 = CXCR1^high^ in mouse) | Mf3 (CD14^low^HLA-DR^high^CD11c^-^)  Mf5 = mainly P5, but could also have some P3  (Mf4 (CD14^high^HLA-DR^high^); in deep mucosa/ submucosa) | CD64^+^CD14^+^CD11c^-^  P1 might be excluded when not gated on HLA-DR^low^ |

**Supplementary Table 3: Comparison of P1-P5 intestinal macrophage subpopulations with previous literature.**
